# Principles of smile design

**DOI:** 10.4103/0972-0707.73387

**Published:** 2010

**Authors:** Mohan Bhuvaneswaran

**Affiliations:** Department of Conservative Dentistry, SRM Dental College and Hospital, SRM University, Chennai, India

**Keywords:** Elements of smile designing, esthetic smile, smile designing, smile proportions

## Abstract

An organized and systematic approach is required to evaluate, diagnose and resolve esthetic problems predictably. It is of prime importance that the final result is not dependent only on the looks alone. Our ultimate goal as clinicians is to achieve pleasing composition in the smile by creating an arrangement of various esthetic elements. This article reviews the various principles that govern the art of smile designing. The literature search was done using PubMed search and Medline. This article will provide a basic knowledge to the reader to bring out a functional stable smile.

## INTRODUCTION

Smile, a person’s ability to express a range of emotions with the structure and movement of the teeth and lips, can often determine how well a person can function in society. Of course, the importance given to a beautiful smile is not new. The search for beauty can be traced to the earliest civilizations; both the Phoenicians (app 800 BC) and Etruscians (app 900 BC) carefully carved animal tusks to simulate the shape, form and hue of natural teeth. It was not until the 18th century that dentistry was recognized as a separate discipline and its various branches were established. Pierre Fauchard (1678–1761) of France, the leader of the movement, together with several colleagues modernized and promoted dentistry and also advocated esthetic practices.[1] This article reviews the various principles that govern the art of smile designing. The literature search was done using pub med search and medline.

## GOALS OF SMILE DESIGNING

The goal of an esthetic makeover is to develop a peaceful and stable masticatory system, where the teeth, tissues, muscles, skeletal structures and joints all function in harmony (Peter Dawson). It is very important that when planning treatment for esthetics cases, smile design cannot be isolated from a comprehensive approach to patient care. Achieving a successful, healthy and functional result requires an understanding of the interrelationship among all the supporting oral structures, including the muscles, bones, joints, gingival tissues and occlusion.[2]

## COMPONENTS OF AN ESTHETIC SMILE

Harmonizing an esthetics smile requires a perfect integration of facial composition and dental composition. The facial composition includes the hard and soft tissues of the face. The dental composition relates more specifically to teeth and their relationship to gingival tissues. A smile design should always include the evaluation and analysis of both facial and dental composition.[3]

### Facial composition

Facial beauty is based on standard esthetic principles that involve proper alignment, symmetry and proportion of face. Analyzing, evaluating and treatment planning for facial esthetics often involve a multidisciplinary approach which could include orthodontics, orthognathic surgery, periodontal therapy, cosmetic dentistry and plastic surgery. Thus, esthetic approach to patient care produces the best dental and facial beauty.[4]

But in our clinical practice, unless and otherwise there is an obvious discrepancy in the face, we restrict our smile makeover to the dental composition only. There are two facial features which do play a major role in the smile design:

1. the interpupillary line and
2. lips.

The interpupillary line should be perpendicular to the midline of the face and parallel to the occlusal plane. Lips are important since they create the boundaries of smile design. If we come across major discrepancies in the above-mentioned two factors, then we have to seriously consider the correction of the facial composition, before we venture into the correction of the dental composition.[5]

**Figure 1:**
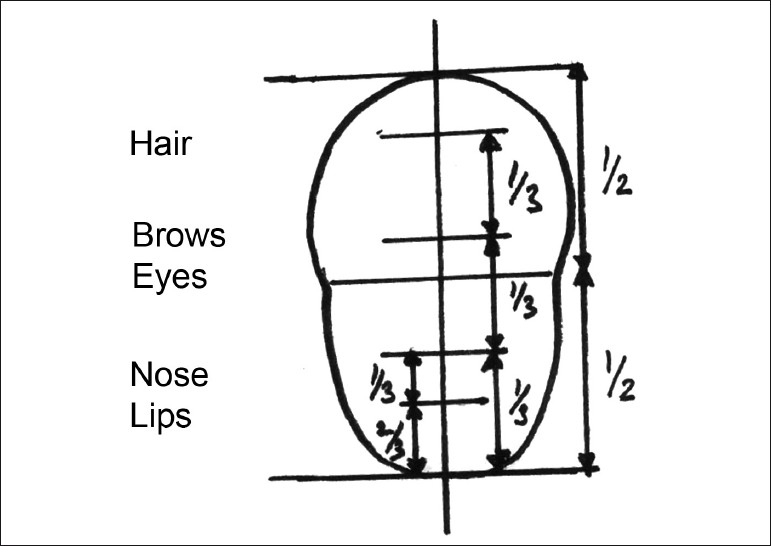
Horizontal dimensions of face

**Figure 2:**
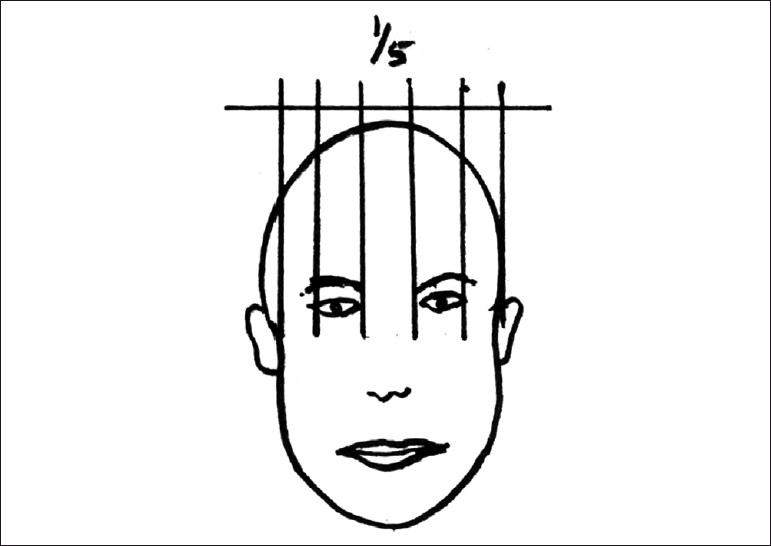
Vertical dimensions of face

In classical terms, the horizontal and vertical dimensions for an ideal face are as follows:

1. Horizontal:
  - The width of the face should be the width of five “eyes”.
  - The distance between the eyebrow and chin should be equal to the width of the face [Figure 1].
2. Vertical:
  - The facial height is divided into three equal parts from the fore head to the eyebrow line, from the eyebrow line to the base of the nose and from the base of the nose to the base of the chin.
  - The full face is divided into two parts, eyes being the midline.
  - The lower part of the face from the base of the nose to the chin is divided into two parts, the upper lip forms one-third of it and the lower lip and the chin two-thirds of it [Figure 2].

The basic shape of the face when viewed from the frontal aspect can be one of the following:

1. Square
2. Tapering
3. Square tapering
4. Ovoid

The lateral profile of an individual can be any one of the following:

1. Straight
2. Convex
3. Concave

These factors play a role in determining the tooth size, shape and the lateral profile; in short, the tooth morphology is dependent on the facial morphology.[6,7]

### Vital elements of smile designing (dental composition)

The vital elements of smile designing include the following:

1. Tooth components
  - Dental midline
  - Incisal lengths
  - Tooth dimensions
  - Zenith points
  - Axial inclinations
  - Interdental contact area (ICA) and point (ICP)
  - Incisal embrasure
  - Sex, personality and age
  - Symmetry and balance
2. Soft tissue components
  - Gingival health
  - Gingival levels and harmony
  - Interdental embrasure
  - Smile line

The role of each of the above-mentioned factors in smile designing is given below.

### Tooth components of smile designing

#### Dental midline

The midline refers to the vertical contact interface between two maxillary centrals. It should be perpendicular to the incisal plane and parallel to the midline of the face. Minor discrepancies between facial and dental midlines are acceptable and, in many instances, not noticeable.[8] However, a canted midline would be more obvious, and therefore, less acceptable. The maximum allowed discrepancy can be 2 mm and sometimes greater than 2 mm discrepancy is esthetically acceptable so long as the dental midline is perpendicular to the interpupillary line. Various anatomical landmarks such as midline of the nose, forehead, chin, philtrum, interpupillary plane can be used as guides to the midline assessment.[9]

The philtrum of the lip is one of the most accurate of these anatomical guide posts. It is always in the center of the face except in surgical, accident or cleft cases. The center of the philtrum is the center of the cupids bow and it should match the papilla between the centrals. If these two structures match and the midline is incorrect, then the problem is usually incisal inclination. If the papilla and philturm do not match, then the problem is a true midline deviation. A midline that does not bisect the papilla is more noticeable than the one that does not bisect the philturm.

To evaluate the midline, one must always consider

1. location and
2. alignment.

Midline should be

- parallel to the long axis of the face: the line angle that forms the contact between the centrals should be parallel to the long axis of the face;
- perpendicular to the incisal plane: the line angle that forms the contact between the centrals should be perpendicular to the incisal plane and
- over the papilla: the midline should drop straight down from the papilla.

A face bow transfer or even a reference stick aligned parallel to the interpupillary plane provides useful information in laboratory communication regarding midline inclination and the possible presence of a canted incisal plane.[10]

Maxillary and mandibular midlines do not coincide in 75% of cases. Therefore, it is not advisable to use the mandibular midline as a reference point for establishing the maxillary midline. Mismatch between maxillary and mandibular midline does not affect esthetics since mandibular teeth are not usually visible while smiling.

#### Incisal lengths (incisal edge positions)

Maxillary incisal edge position is the most important determinant in smile creation because once set, it serves as a reference point to decide the proper tooth proportion and gingival levels. The parameters used to help establish the maxillary incisal edge position are:

1. degree of tooth display,
2. phonetics and
3. patient input

Degree of tooth display: When the mouth is relaxed and slightly open, 3.5 mm of the incisal third of the maxillary central incisor should be visible in a young individual. As age increases, the decline in the muscle tonus results in less tooth display.

Phonetics: Phonetics is a major determinant of the tooth length. In order to determine proper lip, tongue and incisal support and tooth position, it is necessary that the patient sits either erect or stands during the phonetic exercises.[11,12] The various phonetics used are as follows:

- M sound: After pronunciation, the lips return to their normal rest position, allowing evaluation of the amount of the tooth display in rest position.
- E sound: The maxillary incisal edge position should be positioned halfway between the upper and lower lip during the “E” sound.
- F and V sounds: Fricative sounds are produced by the interaction of the maxillary incisal edge with the inner edge of the lower lips’ vermilion border. Thus, fricative sounds help to determine the labiolingual position and length of the maxillary teeth.
- S sound: During pronunciation, the mandibular central incisors are positioned 1 mm behind and 1 mm below the maxillary incisal edge.

Patient input: Intraoral cosmetic preview and provisional restorations help to confirm proper placement of the final incisal edge position. The patient desires must be met as best as possible, provided they do not interfere with the parameters previously discussed.

Correct incisal edge position is crucial because it is related to the pitch of the anterior teeth, labial contours, lip support, anterior guidance, lingual contours and tooth display. The pitch of each anterior tooth is determined by the combination of correct lip support and the lingual labial position of the incisal edge. This location influences anterior guidance and the labial and lingual contours. In short, all these factors play a dominant role in both esthetics and function.

#### Tooth dimensions

Correct dental proportion is related to facial morphology and is essential in creating an esthetically pleasing smile. Central dominance dictates that the centrals must be the dominant teeth in the smile and they must display pleasing proportions. They are the key to the smile. The proportions of the centrals must be esthetically and mathematically correct. The width to length ratio of the centrals should be approximately 4:5 (0.8–1.0); a range for their width of 75–80% of their length is most acceptable. The shape and location of the centrals influences or determines the appearance and placement of the laterals and canines. Various guidelines for establishing correct proportions in an esthetically pleasing smile are

1. golden proportion (Lombardi),
2. recurring esthetic dental proportions (Ward),
3. M proportions (Methot) and
4. Chu’s esthetic gauges.

The important point to be noted here is that it is not the actual size, but instead the perceived size, that these proportions are based on when viewed from the facial aspect (in short, it is the distance between proximal line angles of the teeth).

**Figure 3:**
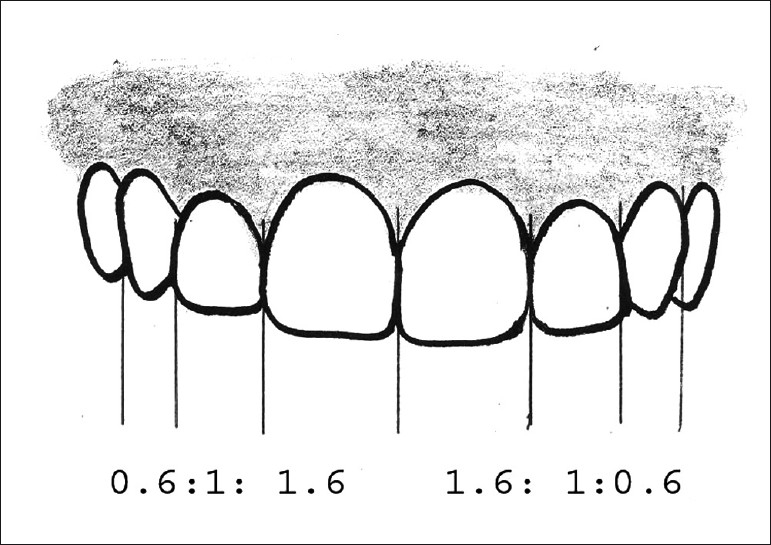
Golden proportion based on apparent width from the frontal view

**Figure 4:**
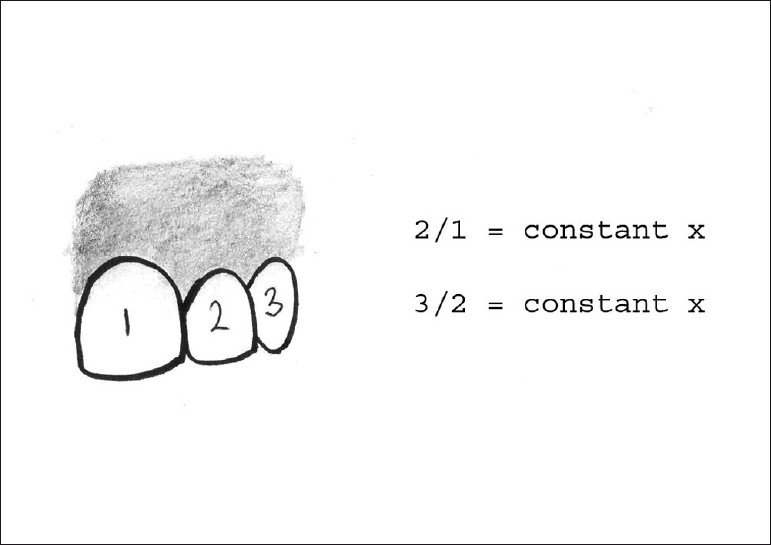
Recurring esthetic dental proportion

1. Golden proportion (Lombardi): When viewed from the facial, the width of each anterior tooth is 60% of the width of the adjacent tooth (mathematical ratio being 1.6:1:0.6) [Figure 3]. It is difficult to apply as patients have different arch form, lip anatomy and facial proportions. Strict adherence to golden proportion calculations limits creativity and this may lead to cosmetic failure.[13]
2. Recurring esthetic dental proportion (Ward): The successive width proportion when viewed from the facial aspect should remain constant as we move posteriorly form midline. This offers great flexibility to match tooth properties with facial proportions [Figure 4].
3. M proportions (Methot): This method compares the tooth width with the facial width using a software. The whole analysis is done in the computer and hence involves more of mathematics rather than artistic analysis.
4. Chu’s esthetic gauges: Dr. Chu’s research supports Levin’s RED concept and refutes the golden proportion. A series of gauges are available to make intraoral analysis easier. The gauges allow for
  - fast, simple analysis and diagnosis of tooth width problems, tooth length problems and gingival length discrepancies;
  - color coding predefines desired tooth proportions, quicker and easier to read than any other instrument;
  - used as a reference guide between clinician and lab technician, hence reduces the incidences of miscommunications errors.

These principles are used as a guide rather than a rigid mathematical formula. Most authors recommend creating harmony and balance by eye via proper adjustment and evaluation of provisionals rather than any formula.[14] The factors guiding individual tooth dimensions are as follows.

Maxillary central incisor: Centrals are the focal point of an esthetic smile and create the central dominance as described earlier. Approximate length of the central should be 10–11 mm and the width is calculated accordingly so that the ratio falls between 75 and 80%

Maxillary lateral incisor: These are the playful part of the smile. They provide individuality, are never symmetrical and influence gender characterization.

Maxillary canine: They play a critical point in creating a pleasing smile as they are

- the junction between the anterior and posterior dental segments; hence, only the mesial half of the canine is visible from the frontal view when the patient smiles;
- support the frontal muscles – the size and characteristic of the buccal corridor is determined by the size, shape and position of the canine and
- canine depicts the personality characterization (masculine: vigorous and aggressive; feminine: delicate and soft).

Also, we have to keep in mind that

- central incisor is wider than the lateral by 2–3 mm and canine by 1–1.5 mm;
- canine is wider than the lateral by 1–1.5 mm and
- canine and central\ are longer than lateral by 1–1.5 mm.

Maxillary bicuspids: They play a very important role for arch design. They should fill the buccal corridor.[15]

**Figure 5:**
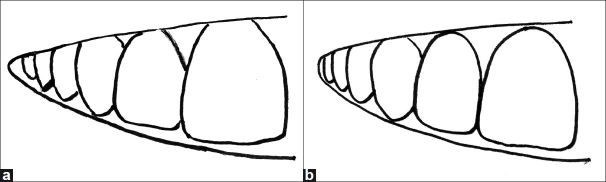
a) Insufficiently developed buccal corridor, b) properly developed buccal corridor

Buccal corridor refers to dark space (negative space) visible during smile formation between the corners of the mouth and the buccal surfaces of the maxillary teeth [Figure 5a and b]. Its appearance is influenced by

1. the width of the smile and the maxillary arch,
2. the tone of the facial muscles,
3. the positioning of the labial surface of the upper premolars,
4. the prominence of the canines particularly at the distal facial line angle and
5. any discrepancy between the value of the premolars and the six anterior teeth.

Arch form has a direct influence on the buccal corridor.[16] The ideal arch is broad and conforms to a U shape. A narrow arch is generally unattractive. The unattractive, negative space should be kept to a minimum. This problem can be solved or minimized by restoring the premolars. The buccal corridor should not be completely eliminated because a hint of negative space imparts to the smile a suggestion of depth.

Ultimately, there is no formula for anterior esthetics; instead, the final esthetics is a combination of

1. tooth proportion guide lines,
2. patient’s own perception,
3. cultural and social influences,
4. dentist artistic influences and
5. effective communication with laboratory.

#### Zenith points

**Figure 6:**
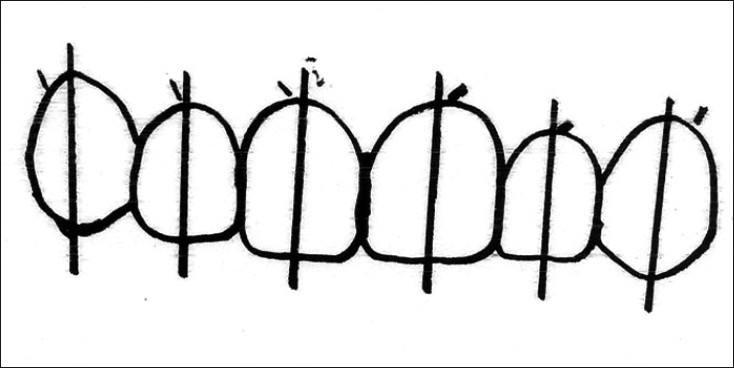
Zenith points and its relation to midline

Zenith points are the most apical position of the cervical tooth margin where the gingiva is most scalloped. It is located slightly distal to the vertical line drawn down the center of the tooth. The lateral is an exception as its zenith point may be centrally located[17] [Figure 6]. Establishing the proper location of zenith points is a critical step in alteration of mesial and distal dimensions,

1. closure of diastema: move the zenith points to
2. provide the illusion of bodily movement and reduce exaggerated triangular form and
3. correction of tooth angulation.

#### Tooth inclinations

Axial inclination compares the vertical alignment of maxillary teeth, visible in the smile line, to central vertical midline. From the central to the canine, there should be natural, progressive increase in the mesial inclination of each subsequent anterior tooth. It should be least noticeable with the centrals and more pronounced with the laterals and slightly more so with the canines. If the incisal plane is canted, the axial inclination of the anterior teeth and the midline itself, if it is at right angle to the incisal plane, will be correspondingly incorrect.[15]

The evaluation of axial inclination can be done on a photograph of the anterior teeth in a frontal view. A line is sketched on each tooth from the middle of the incisal edge through the midline of the tooth at its gingival interface. Axial inclination can also refer to the degree of tipping in any plane of reference. The guide for labiolingual inclination is as follows:

1. Maxillary central incisor – positioned vertically or slightly labial
2. Maxillary lateral incisor – cervical is tucked in, incisal edge inclined slightly labially
3. Maxillary canine – cervical area positioned labially, cusp tip lingually angulated

#### Interdental contact area and point

1. Interproximal contact area (ICA):
  - It is defined as the broad zone in which two adjacent teeth touch.
  - It follows the 50:40:30 rule in reference to the maxillary central incisor [Figure 7].
  - The increasing ICA helps to create the illusion of longer teeth by wider and also extend apically to eliminate black triangles.
2. Interproximal contact point (ICP):
  - It is the most incisal aspect if the ICA.
  - As a general rule, the ICP moves apically, the further posterior one moves from the midline [Figure 8].

**Figures:**
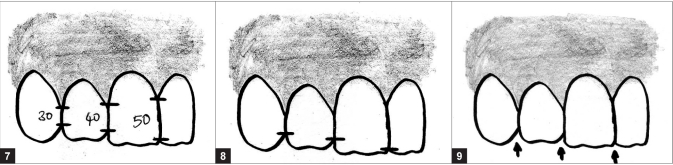
(7) ICAs – 50:40:30 rule, (8) ICPs – moves appically as we move from central to canine, (9) Incisal embrasure – increase in size and depth from central to canine

#### Incisal embrasures

The incisal embrasures should display a natural, progressive increase in size or depth from the central to the canine. This is a function of the anatomy of these teeth and as a result, the contact point moves apically as we proceed from central to canine [Figure 9]. The contact points in their apical progression should mimic the smile line.[16] Failure to provide adequate depth and variation to the incisal embrasure will

1. make the teeth appear too uniform and
2. make the contact areas too long and impart to the dentition a box like appearance. The individuality of the incisors will be lost if their incisal embrasures are not properly developed.

Also, if the incisal embrasures are too deep, it will tend to make the teeth look unnaturally pointed. As a rule, a tooth distal to incisal corner is more rounded than its mesio incisal corner.

#### Sex, age and personality

Minor differences in the length, shape and positioning of the maxillary teeth allow for dramatic characterization.[18]

- Age – maxillary central incisor
  Youthful teeth: unworn incisal edge, defined incisal embrasure, low chroma and high value
  Aged teeth: shorter; so less smile display, minimal incisal embrasure, high chroma and low value
- Sex – maxillary incisors
  Female form: round smooth, soft delicate
  Male form: cuboidal, hard vigorous
- Personality – maxillary canine
  Aggressive, hostile angry: pointed long “fangy” cusp form Passive, soft: blunt, rounded, short cusp form

#### Symmetry and balance

Symmetry is the harmonious arrangement of several elements with respect to each other. Symmetrical length and width is most crucial for the centrals. It becomes less absolute as we move further away from the midline

- Static symmetry: mirror image, maxillary central incisors
- Dynamic symmetry: two objects very similar but not identical. Playing with perfect imperfection in the laterals and canines allows for a more vital, dynamic, unique and natural smile.[16]

Balance is observed as the eyes move distally from the midline, so that both the right and left sides of the smile are well balanced.

### Soft tissue component of smile design

#### Gingival health

The gingiva acts as the frame for the teeth; thus, the final esthetic success of the case is greatly affected by the gingival health. It is of paramount importance that the gingival tissues are in a complete state of health prior to the initiation of any treatment.[19] Healthy gingiva is usually

1. pale pink in color, stippled, firm and it should exhibit a matte surface;
2. located facially – 3 mm above the alveolar crestal bone and
3. located interdentally – 5 mm above the intercrestal bone papilla should be pointed and should fill the gingival embrasure right up to the contact area.

#### Gingival level and harmony

Establishing the correct gingival levels for each individual tooth is the key in the creation of harmonious smile. The cervical gingival height (position or level) of the centrals should be symmetrical. It can also match that of the canines. It is acceptable for the laterals to display the same gingival level. However, the resultant smile may be too uniform and it is preferable to exhibit a rise and fall in the soft tissue by having the gingival contour over the laterals located toward the incisal compared to the tissue level of the centrals and canines [Figure 10]. The gingival margin of the lateral incisor is 0.5–2.0 mm below that of the central incisors. The least desirable gingival placement over the laterals is for it to be apical to that of the centrals and or the canines.[16]

**Figures:**
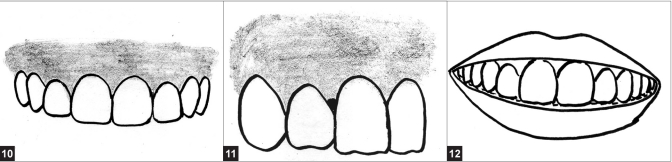
(10) Ideal gingival level – centrals and canines same level and laterals cervical to them, (11) Interdental embrasure – showing black triangle, (12) Smile line that follows the superior border of the lower lip

The gingival shape on the mandibular incisors and the maxillary laterals should exhibit a symmetrical half oval or half circular shape. The maxillary centrals and canines should exhibit a gingival shape that is more elliptical. Thus, as mentioned earlier, the gingival zenith is located distal to the long axis of the maxillary centrals and canines and coincides along the long axis of the maxillary lateral incisors.[17,20]

#### Interdental embrasure (cervical embrasure)

The darkness of the oral cavity should not be visible in the interproximal triangle between the gingiva and the contact area. If the most apical point of the restoration is 5 mm or less from the crest of the bone, then black triangles will be avoided. At times, this will require long contact area that will be extended toward the cervical. This will encourage the formation of a healthy, pointed papilla instead of the blunted tissue form that often accomplishes a black triangle[21] [Figure 11]. Conversely, an improperly developed cervical embrasure that involves overextended, bulky restorations will result in an improper emergence profile and swollen and inflamed gingival tissues.[22]

#### Smile line

Smile line refers to an imaginary line along the incisal edges of the maxillary anterior teeth which should mimic the curvature of the superior border of the lower lip while smiling. Another frame of reference for the smile line suggests that the centrals should appear slightly longer or, at least, not any shorter than the canines along the incisal plane. This approach is particularly useful in cases of lip symmetry or extreme lip curvature during smile formation [Figure 12]. Reverse smile line or inverse smile line occurs when the centrals appear shorter than the canines along the incisal plane.

Lip line should not be confused with the smile line. It refers to the position of the inferior border of the upper lip during smile formation and thereby determines the display of tooth or gingiva at this hard and soft tissue interface. Under ideal conditions, the gingival margin and the lip line should be congruent or there can be a 1–2 mm display of the gingival tissue. Showing 3–4 mm or more of the gingiva (gummy smile) often requires cosmetic periodontal recontouring to achieve an ideal result.[23]

Finally, the individual tooth morphology has to mimic nature, once all the above-mentioned factors are fulfilled.[7] Also, the appropriate shade selection has to be done to bring out all the hard work of our smile design. Shade selection must be customized for each individual. It should be natural and polychromatic. The body of the tooth can be fairly uniform in color but the gingival third should be noticeably richer in chroma. The chroma should also increase from central to the canine, canine having a higher chroma.[24]

## CONCLUSION

It is vivid from the above discussion that the smile we create should be esthetically appealing and functionally sound too. It is our duty to carefully diagnose, analyze and deliver the best to our patients, taking into account all of the discussed factors. The smile designing done by us has to be as conservative as possible unlike the past. Our aim has to be less reduction of tooth structure and greater esthetics and durability. This simply means that cosmetic dentistry has to be a multispecialty branch, wherein all treatments like orthodontics, periodontics, surgical procedures have to be performed whenever deemed necessary.
